# Effects of South-to-North Water Diversion Project Cascade Dams on Riparian Vegetation Along the Middle and Lower Reaches of the Hanjiang River, China

**DOI:** 10.3389/fpls.2022.849010

**Published:** 2022-02-22

**Authors:** Jiao Yang, En-Hua Li, Chao Yang, Ying Xia, Rui Zhou

**Affiliations:** ^1^Key Laboratory for Environment and Disaster Monitoring and Evaluation of Hubei Province, Innovation Academy for Precision Measurement Science and Technology, Chinese Academy of Sciences, Wuhan, China; ^2^University of Chinese Academy of Sciences, Beijing, China

**Keywords:** riparian zone, vegetation types, cascade dams, two-way indicator species analysis (TWINSPAN), RDA (redundant analysis), Hanjiang River

## Abstract

The influence of the construction of dams for water diversion on the ecological environment has attracted recent widespread attention. Over time, dams have emerged as one of the most important factors affecting the vegetation along the riparian zones of rivers. To elucidate the effects of cascade dams on riparian vegetation along the middle and lower reaches of the Hanjiang River, we examined riparian vegetation types upstream and downstream from dams. A total of 14 sample sites and 131 quadrats perpendicular to the river were investigated in June 2019, and 14 sample sites and 134 quadrats were investigated in October 2019. The riparian vegetation was divided into 15 (in June) and 11 (in October) vegetation types by two-way indicator species analysis (TWINSPAN). Significant differences were found between the vegetation types upstream and downstream of dams. Redundancy analysis (RDA) showed that soil moisture content, distance from the water, altitude and soil total nitrogen (TN) were the main environmental factors affecting plants distributions, and soil moisture content was the main factor affecting the zonal distribution of vegetation. By analyzing the impact of cascade dams on the hydrological regime, we found that the construction of cascade dams led to the differentiation of vegetation types upstream and downstream of the dam, and the riparian habitats were fragmented by these dams. This study provides both an important reference for the protection of riparian vegetation and riparian ecosystems and a basis for the management and restoration of river ecosystems after the construction of cascade dams.

## Introduction

Riparian ecosystems are the transition zones from aquatic ecosystem to terrestrial ecosystems and one of the most diverse, rapidly changing and complex habitats on earth (Naiman and Décamps, 1997; Chen et al., 2020). Riparian vegetation is an important component of riparian ecosystems (Stromberg, 2001). Riparian vegetation is the primary producer in a riparian ecosystem and plays important functions in energy flow, information flow and biological flow between aquatic and terrestrial ecosystems (Artini et al., 2021). At the same time, vegetation in the riparian zone can control soil erosion and non-point source pollutants in the riparian zone and has unique environmental functions in the whole river ecosystem (Story et al., 1953). Riparian ecosystems are generated and maintained by geographic variation in stream processes and fluvial disturbance regimes, which largely reflect regional differences in climate and geology (Bendix and Hupp, 2000). Natural flow variability is the primary driver of riverine ecosystem functions and structures (Wang et al., 2015). Natural disturbances, such as floods and droughts, are integral components of riparian ecosystems (Lytle and Poff, 2004; Rivaes et al., 2017). River water transmission has a positive effect on the migration of vegetation communities along river bank zones, and vegetation responds to climate change and land use changes through river water migration (Nilsson et al., 2010).

With growth in both water stress and the demand for energy as well as increases in the ability of people to exploit resources and prevent natural disasters, more dams have been constructed (Graham et al., 2019; Maavara et al., 2020). More than 70,000 large dams have been built worldwide; approximately 70% of the world’s rivers are intercepted by large reservoirs, and this number will continue to increase in the foreseeable future (Kummu and Varis, 2007; Maavara et al., 2015). Dams can cause changes in physical, chemical and biological aspects of the environment (Castro et al., 2021). They have not only brought social and economic value to human beings, but have also changed the natural river ecological environment (Humborg et al., 1997; Bednarek and Hart, 2005; Domingues et al., 2014; Chen et al., 2021). From an ecological perspective, the construction and operation of a dam divides a previously continuous river ecosystem into discontinuous and relatively independent habitats, which has negative ecological significance worldwide and has a profound impact on the entirety of each individual river ecosystem (Poff et al., 2007). Dams change the natural river hydrological conditions, leading to a rise of the water level upstream of a dam and a change in the timing, magnitude and frequency of flooding downstream (Shiau and Wu, 2013). These issues have resulted in the differentiation of plant composition and community types in river riparian zones (Nilsson et al., 2010). Dams have been demonstrated to cause habitat heterogeneity in riparian zones, resulting in reduced species and functional diversity, the invasion of alien species and the loss of native species (Nilsson et al., 2005; Merritt and Wohl, 2010). Large dams can change the riparian vegetation both upstream and downstream, posing substantial threats to native river biodiversity (Wei et al., 2008). As society often pays attention to environmental problems, the influence of dam construction in key areas on the vegetation in the riparian zone has always been a key topic in the study of river ecosystems (Fu and Burgher, 2015). Studying the response of riparian vegetation to dams can benefit both river ecological management and restoration of riparian vegetation, which is of great significance to the protection of the integrity of river ecosystems (Takahashi and Nakamura, 2011).

The South-to-North Water Diversion Project is aimed at alleviating severe water shortages in Northern China and is the largest water diversion project in the world (Yao et al., 2019). The Hanjiang River, the largest tributary of the Yangtze River, is an important channel for the middle route of China’s South-to-North Water Diversion Project (Zhao et al., 2021). Three cascade dams have been built in the main stream of the middle and lower reaches of the Hanjiang River, and three cascade dams are planned to be built in the future (Wang et al., 2015). Since the upper Danjiangkou Dam was upgraded in 2005 and the South-to-North Water Diversion Project began to supply water in 2014, the impact on the ecological environment of the middle and lower reaches of the Hanjiang River has been incalculable (Gao et al., 2020; Yu et al., 2020). The published studies on the ecological environment related to this project have mainly focused on the area upstream of the Danjiangkou Dam, and there have been fewer studies on the middle and lower reaches (Li et al., 2012). Published studies on the middle and lower reaches of the Hanjiang River have mainly focused on ecohydrological conditions, sediment regimes, characteristics of floodplain sediments, diatom blooms and other phenomena related to the changes in physical environment (Tian and Zhou, 2008; Lu et al., 2012; Yang Q. et al., 2012; Wang et al., 2015). Compared to a single dam, the impact of cascade hydropower dam construction on watershed ecosystems is more serious owing to its multiple instances of obstruction and interception (Rodríguez-Pérez et al., 2021). Knowledge of the state of existing vegetation is a prerequisite for all conservation efforts. However, there are few studies on the effects of cascade dams on vegetation in the middle and lower reaches of the Hanjiang River. In this study, based on an abundance of field data, the characteristics of vegetation types in the riparian zones and the differences in plant communities between the areas upstream and downstream of the dams were assessed. Additionally, the key factors affecting vegetation types in the riparian zone and the law of plant community succession were discussed.

## Materials and Methods

### Study Area

**FIGURE 1:**
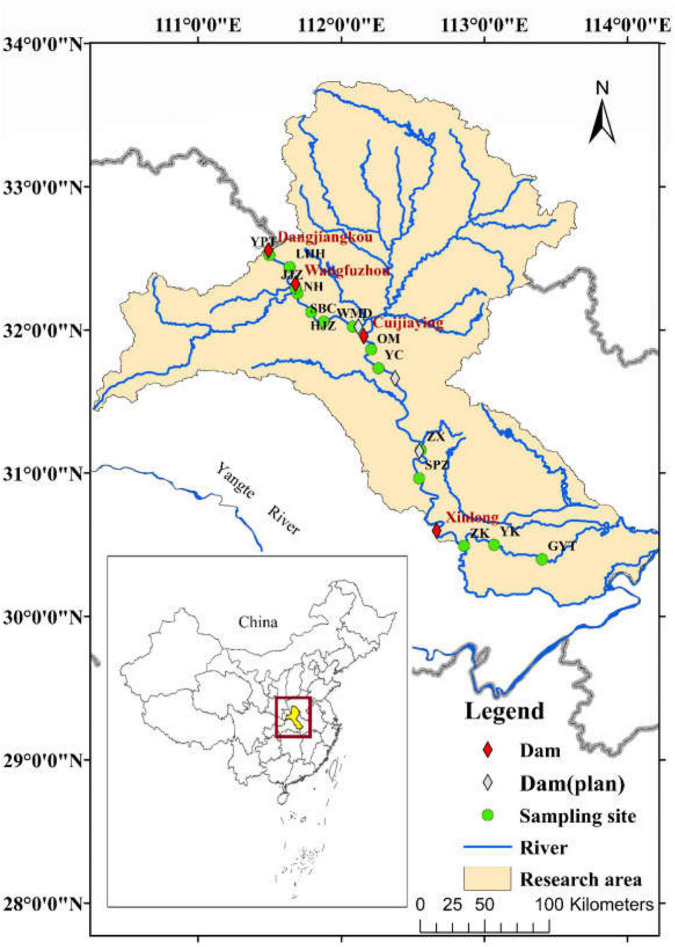
Location of the study area and sampling sites. The sampling sites were named after abbreviations of the local place names: YPT, Yangpitan (B); LHH, Lihuahu (B); JJZ, Jiangjiazhou (B); NH, Nanhu (I); HJZ, Hujiazhou (B); SBC, Shanbiancun (I); WMD, Wumingdao (I); OM, Oumiao (I); YC, Yicheng (B); ZX, Zhongxiang (I); SPZ, Shipaizhen (I); ZK, Zekou (B); YK, Yuekou (B); GYT, Guanyintang (B). Following each sample site, B in parentheses indicates a beach location, and I indicates a central island of the river.

The Hanjiang River, the largest tributary of the Yangtze River, originates at the southern foot of the Qinling Mountains, Shanxi Province, China. The Hanjiang River has a total length of 1,577 km, a basin area of 174,300 km^2^, and an altitude difference from source to mouth of 1,964 m. This river flows through Shanxi and Hubei Provinces, joining the Yangtze River in Wuhan, Hubei Province. The Hanjiang River is situated in the East Asian subtropical monsoon climate zone. This climate zone has four distinct seasons, a mild climate, an average temperature of 16°C, a frost-free period of more than 250 days, and a mean precipitation of 700–1300 mm, which is concentrated from May to October, accounting for 70–80% of annual rainfall (Wang et al., 2015; Guo et al., 2016). The Hanjiang basin is situated between the Daba Mountain and the southern piedmont of the Qinling Mountains, and the area of the mountains and hills accounts for approximately 83% of the total drainage area (Xu et al., 2011). The upper reaches of the Hanjiang River are situated upstream of Danjiangkou, alternating between basins and canyons with a length of about 925 km; the middle reaches of the river are situated between Danjiangkou and Zhongxiang, a hilly and valley basin with a length of about 270 km. The middle reaches of the river valley are wide, forming numerous sandy beaches. Due to the slow flow in the section, a large amount of river sand accumulates, resulting in unstable deposition and erosion of the riverbed (Zhang et al., 2020). The river ranges from 0.3–0.4 to 2–3 km wide between the dry and flood seasons, respectively, and it is 5–6 km wide at its widest point. The lower reaches of the river are situated downstream of Zhongxiang and flow through the Jianghan Plain with a length of about 382 km. This section of the river is slow, and the channel narrows here, becoming less than 0.2 km wide near the estuary (Xu et al., 2017). The Hanjiang River is an important passage for the middle route of the South-to-North Water Diversion Project in China. Our study was mainly conducted across the middle and lower reaches of the Hanjiang River (Figure 1).

### Survey Sites and Vegetation Sampling

**FIGURE 2:**
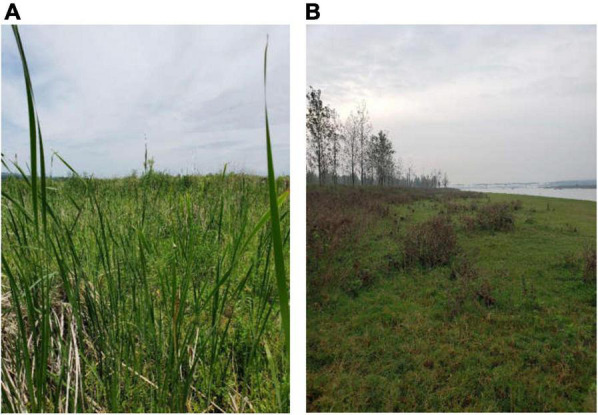
**(A)** Typical riparian vegetation upstream of a dam [the sample site of Lihuahu (LHH), June 06, 2019]; **(B)** typical riparian vegetation downstream of a dam [the sample site of Nanhu (NH), October 23, 2019].

Field vegetation investigations of the middle and lower reaches of the Hanjiang River were undertaken in the early summer of 2019 (June) and the autumn of 2019 (October), when the riparian zones were emerged extensively and the dominant plants were in their flowering and fruiting period. Based on the field investigations, sampling sites were established in 14 typical areas, all of which were natural riparian zones (including central islands) upstream and downstream of the dams with relatively less disturbance from human activities (Figures 1, 2).

Based on several times field investigations, we found that the characteristics of vegetation were distributed in transects along the water gradient, and species composition and community structure relatively consistent in each transect, thus each sampling site was divided into 2–4 transects. Since herbaceous species were the main natural species in our study area, woody species only existed in the high part and were very rare, we only investigated herbaceous plants in the sample sites. Three to six 1 m × 1 m quadrats were situated in each transect. There were 14 sampling sites in June (2019) and October (2019), with 131 and 134 quadrats, respectively. In each quadrat, the species coverage of each vegetation, the total coverage of the quadrat (based on the sum of the crown areas), the height and the number of each vegetation were recorded (Li et al., 2018). As per the method described by Xiu et al. (2015), the number of stems was regarded as the number of plant individuals. Plants that had not completely died were also included in the plant surveys. For each transect, names of species outside the quadrat were also recorded^1^.

For each quadrat, altitude, latitude and longitude were recorded using an ATK-S822013 GPS receiver (South Surveying & Mapping Technology Co., Ltd, Guangzhou, China). Soil moisture data for each quadrat was recorded at a depth of 10 cm using a soil hygrometer HD2 (IMKO Micromodultechnik, Germany). The distance from the water was calculated based on the longitude and latitude of the quadrat and the riverside along the same transect line (Manolaki and Papastergiadou, 2013). Soil samples were collected from the topsoil (0–10 cm depth). All soil samples were naturally air-dried, crushed and screened using a 0.088 mm sieve for fine and coarse soil components (Xiu et al., 2015). Total nitrogen (TN) content, total phosphorus (TP) content and soil organic matter (SOM) were determined using standard methods (Qian et al., 1990; Frangipane et al., 2010).

### Statistical Analysis

The species importance value (IV) for the different plants in each quadrat were calculated as follows (Curtis and Mcintosh, 1951; Li et al., 2018):


IV=(RF+RH+RC)/3

Here, RF is the relative frequency, RH is the relative height, and RC is the relative coverage.

A plant community matrix consisting of each species IV with a frequency ≥5% in the quadrat was selected (Chambers et al., 2004). Two-way indicator species analysis (TWINSPAN) was used to classify the samples and plant species (Li et al., 2012). According to the principles of plant community classification and field investigation, the vegetation types of indicator species and the dominant species were analyzed. The analysis was performed using the computer program PC-ORD (version 5) (Mccune and Mefford, 1999).

Soil properties of the different quadrats were compared using analysis of one-way variance (ANOVA), and a *post-hoc* Tukey’s test was conducted to determine significant groupings (*P* < 0.05) (Wang et al., 2017). Descriptive statistical parameters were calculated and significance tests were conducted using SPSS (version 22, IBM Corp., Armonk, NY, United States). Redundancy analysis (RDA) was performed to clarify relationships between quadrats and environmental factors (Xiu et al., 2015). RDA analysis was undertaken using R (version 3.6.1, R Foundation for Statistical Computing, Vienna)^2^. Data pretreatment and analysis were completed using Excel 2010 (Microsoft Corp., Redmond, WA, United States).

## Results

### Vegetation Type Classification

In June and October, 110 species belonging to 94 genera and 43 families and 120 species belonging to 99 genera and 44 families, respectively, were found in the middle and lower reaches of the Hanjiang River. The TWINSPAN analysis classified 131 quadrats into 15 vegetation types in June and 134 quadrats into 11 vegetation types in October (Table 1). In June, the first transect near the water was mainly comprised of communities of *Polygon fugax*, *Paspalum distichum*, *Typha angustifolia*, *Cynodon dactylon*, *Phalaris arundinacea*, and *Erigeron canadensis* in the middle and lower reaches of the Hanjiang River. In the second transect and above, the areas were mainly composed of *C. dactylon*, *Artemisia argyi*, *Imperata cylindrical*, *Bromus japonicus*, and *Phragmites australis*. In October, the first transect was mainly comprised of communities of *T. angustifolia*, *P. arundinacea*, *P. distichum*, *P. australis*. The second transect and above was mainly comprised of communities of *C. dactylon*, *P. arundinacea*, *A. argyi*, *Saccharum arundinaceum, I. cylindrica*, *P. distichum*, and *P. australis*. To sum up, there was no significant difference in vegetation types between October and June in different transects except among some seasonal annual herbs.

**TABLE 1:** Vegetation types in the riparian zone along the middle and lower reaches of the Hanjiang River.

*No.*	*Vegetation types*	*Coverage mean + S.D*	*Sample name (sample site followed by number)*		*Per. T (%)*	*Habitat*
			Middle	Lower		
***June***						
*I*	*Cynodon dactylon*	88.93 ± 18.26	HJZ2, HJZ3, YC2	ZK1, GYT2	11.45	Downstream
*II*	*C. dactylon*, *Erigeron canadensis*	98.60 ± 0.89		GYT1, SPZ1, YK3, YK1, YK2	8.40	Downstream
*III*	*Bromus japonicus*	76.80 ± 23.24	JJZ2, JJZ3, NH4, OM3, YC3		11.45	Downstream
*IV*	*Polypogon fugax*	78.83 ± 12.70	JJZ1, SBC1		4.58	Downstream
*V*	*Phragmites australis*	85.00 ± 16.32	ZX2, ZX3	ZK2, SPZ2	6.87	Downstream
*VI*	*P. australis*, *Phalaris arundinacea*	90.33 ± 11.25	SBC2, ZX2	YK2, ZK2	4.58	Downstream
*VII*	*P. arundinacea*	97.08 ± 2.64	YPT1, NH2, NH3, OM2	SPZ1, GYT1	9.16	Downstream
*VIII*	*Artemisia argyi*	77.30 ± 20.13	SBC2, SBC3, WMD2, OM4		7.63	Downstream upstream
*IX*	*Imperata cylindrica*	92.75 ± 9.07	YPT3, YPT4, LHH2		6.87	Downstream upstream
*X*	*Rumex dentatus*, *Daucus carota*	66.00 ± 3.61	OM1		2.29	Downstream
*XI*	*Hemarthria sibirica*	95.00 ± 5.57	YPT2		2.29	Downstream
*XII*	*Equisetum arvense*, *I. cylindrica*	97.50 ± 2.43	LHH3, WMD2		4.58	Upstream
*XIII*	*Paspalum distichum*	98.80 ± 0.45	NH1, HJZ1, YC1, SBC2, ZX1	ZK3	12.98	Downstream
*XIV*	*Melilotus officinalis*	95.33 ± 3.06		ZK4	2.29	Downstream
*XV*	*Typha angustifolia*	91.83 ± 10.23	WMD1, LHH1		4.58	Upstream
***October***						
*I*	*C. dactylon*	88.60 ± 11.02	NH2, NH3, HJZ3, JJZ3, JJZ2, YC2, YC3, SBC2, SBC3, OM3, ZX2	GYT2, GYT3, YK2, YK3	29.85	Downstream
*II*	*C. dactylon*, *E. canadensis*	97.00 ± 1.73		ZK3	2.24	Downstream
*III*	*P. arundinacea*	83.40 ± 24.19	OM2, YPT1, ZX2	SPZ3, ZK2, ZK4, YK1, GYT1	12.69	Downstream
*IV*	*Saccharum arundinaceum*	76.67 ± 21.20	LHH3, OM3		2.24	Downstream upstream
*V*	*A. argyi*	95.33 ± 4.04	WMD3		2.24	Upstream
*VI*	*I. cylindrica*	95.00 ± 5.47	YPT3, LHH3, WMD2, LHH2		8.21	Downstream upstream
*VII*	*P. distichum*	66.06 ± 24.01	JJZ1, NH1, SBC1, OM1, ZX1, YC1, HJZ1, HJZ2	SPZ1, SPZ2, ZK1, ZK4, YK2	26.12	Downstream
*VIII*	*P. australis*	53.13 ± 27.21	YPT1, ZX3	ZK1, SPZ2	5.97	Downstream
*IX*	*T. angustifolia*	88.14 ± 8.90	LHH1, WMD1		5.22	Upstream
*X*	*H. sibirica*	86.25 ± 17.58	YPT2	ZK1	2.99	Downstream
*XI*	*I. cylindrica*, *Aster tataricus*	83.67 ± 18.88		YK4	2.24	Downstream

*Per. T, percent total numbers of quadrats sampled for each vegetation; YPT, Yangpitan; LHH, Lihuahu; JJZ, Jiangjiazhou; NH, Nanhu; HJZ, Hujiazhou; SBC, Shanbiancun; WMD, Wumingdao; OM, Oumiao; YC, Yicheng; ZX, Zhongxiang; SPZ, Shipaizhen; ZK, Zekou; YK, Yuekou; GYT, Guanyintang. Middle, the middle reaches of the Hanjiang River; Lower, the lower reaches of the Hanjiang River. Upstream, upstream of the dams; Downstream, downstream of the dams.*

In June, the middle reaches of the Hanjiang River were mainly comprised of communities of *P. fugax*, *P. distichum*, and *T. angustifolia* in the first transect, while the second transect and above was mainly composed of xerophytes such as *C. dactylon*, *B. japonicus*, *A. argyi*, *I. cylindrica*, *P. australis*. The lower reaches of the river were mainly comprised of some xerophytes such as *C. dactylon*, *E. canadensis* both in all transects, while the second transect and above was mainly composed of some emergent aquatic plant such as *P. australis*, *P. arundinacea*, *P. distichum*. In October, except the communities in Wumingdao (WMD) and Lihuahu (LHH), most of the communities were both present in the middle and lower reaches.

In June, there were four vegetation types in the reservoir area upstream of the dams and thirteen vegetation types in the area downstream of the dams, and of these, three vegetation types spanned the dams. In October, there were four vegetation types upstream of the dams and nine vegetation types downstream of the dams, and of these, two vegetation types spanned the dams. The vegetation types upstream of the dams were stable both in June and October. The first transect near the water was mainly composed of large emergent plants, such as *T. angustifolia*. The transect upland of the second transect was mainly composed of perennial xerophytes, such as *I. cylindrica* and *A. argyi*. The first transect downstream of the dams was different from that upstream of the dams, it was mainly composed of perennial wetland herbs, such as *P. distichum* and *P. arundinacea*. Plant communities in some areas were seasonal. The upland areas downstream of the dams were not much different from those upstream of the dams and were dominated by xerophytes such as *I. cylindrica*.

### Redundancy Analysis Gradient Analysis

The RDA indicated that the first four axes of ordination explained 76.4 and 65.1% of the variance in environmental data corresponding to quadrats (species) for 131 and 134 quadrats in June and October, respectively (Table 2). Permutation test results on all axes were significantly correlated (*P* < 0.01), respectively. Along the first axis, the contents of soil TN, soil organic matter (SOM) and altitude (Alt) gradually decreased from left to right. The change in total phosphorus (TP) along this axis was not obvious. The second axis reflected that the effects of variation in soil moisture (Moist) decreased gradually from the water’s edge to the highland in the riparian habitat, and such a pattern showed the opposite trend in the distance from the water. These results were consistent with the results of one-way ANOVA of environmental factors (Table 3). Moist and distance from water (Dis) were two key environmental factors, followed by Alt, TN, and SOM. By comparing environmental factors between groups, we found significant differences in environmental factors upstream and downstream of the dams. TN, SOM, and Moist in the first transects upstream of the dams were significantly higher than the sampling sites downstream of the dams. The construction of the dams resulted in environmental differences upstream and downstream of the dams.

**TABLE 2:** Eigenvalues and species-environment correlations of redundancy analysis (RDA) axes of quadrats along the middle and lower reaches of the Hanjiang River.

	June (131 quadrats)				October (134 quadrats)			
	Axis 1	Axis 2	Axis 3	Axis 4	Axis 1	Axis 2	Axis 3	Axis 4
Eigenvalues	0.4992	0.1865	0.0781	0.0005	0.4471	0.1363	0.0679	0.0000
Species–environment correlations	0.8960	0.8762	0.7621	0.8811	0.8317	0.7983	0.6968	0.5408
Cumulative percentage of variance in species–environment correlations (%)	49.92	68.58	76.38	76.44	44.71	58.33	65.12	65.12
Permutation test results on all axes	*pseudo-F* = 6.1, *P* = 0.002				*pseudo-F* = 6.1, *P* = 0.002			

**TABLE 3:** Mean values (± standard deviations) of environment factors along with one-way ANOVA comparing clustered vegetation groups.

Type	Physicochemical factors					
	**TN**	**TP**	**SOM**	**Moist**	**Alt**	**Dis**
**June**						
I	0.12 ± 0.08^ac^	0.45 ± 0.17^a^	0.04 ± 0.02^a^	7.38 ± 2.34^b^	28.41 ± 16.22^b^	39.32 ± 19.81^b^
II	0.07 ± 0.07^a^	0.84 ± 0.11^c^	0.02 ± 0.00^a^	19.09 ± 6.08^a^	13.20 ± 3.98^c^	4.28 ± 2.38^c^
III	0.17 ± 0.11^a^	0.42 ± 0.08^a^	0.04 ± 0.02^a^	11.71 ± 10.58^a^	45.40 ± 12.29^a^	91.59 ± 59.32^a^
IV	0.24 ± 0.16^a^	0.40 ± 0.00^a^	0.04 ± 0.00^a^	22.25 ± 2.90^a^	47.76 ± 7.10^a^	11.60 ± 7.17^b^
V	0.03 ± 0.00^a^	0.56 ± 0.15^a^	0.02 ± 0.01^a^	7.21 ± 3.12^b^	17.51 ± 3.21^b^	27.71 ± 13.31^b^
VI	0.06 ± 0.06^a^	0.51 ± 0.16^a^	0.03 ± 0.02^a^	14.07 ± 3.38^a^	16.29 ± 13.46^bc^	19.64 ± 7.34^b^
VII	0.28 ± 0.22^a^	0.51 ± 0.16^a^	0.06 ± 0.03^a^	19.37 ± 13.19^a^	45.50 ± 16.18^a^	40.30 ± 52.62^b^
VIII	0.26 ± 0.13^a^	0.49 ± 0.07^a^	0.06 ± 0.02^a^	6.23 ± 1.75^b^	42.63 ± 2.04^a^	31.23 ± 5.50^b^
IX	0.80 ± 0.08^b^	0.50 ± 0.06^a^	0.09 ± 0.09^b^	10.65 ± 2.46^b^	65.14 ± 3.18^d^	23.82 ± 5.97^b^
X	0.10 ± 0.01^a^	0.35 ± 0.01^b^	0.04 ± 0.00^a^	37.81 ± 17.20^a^	30.23 ± 0.10^b^	10.59 ± 1.43^bc^
XI	0.30 ± 0.01^a^	0.42 ± 0.01^a^	0.04 ± 0.00^a^	15.68 ± 3.20^ab^	63.03 ± 0.33^d^	7.54 ± 0.13^b^
XII	0.52 ± 0.08^a^	0.53 ± 0.00^a^	0.08 ± 0.01^b^	5.25 ± 4.29^b^	50.17 ± 14.02^ad^	26.33 ± 10.26^b^
XIII	0.29 ± 0.20^a^	0.43 ± 0.06^a^	0.05 ± 0.03^a^	41.10 ± 19.08^a^	49.05 ± 2.24^a^	11.92 ± 6.02^b^
XIV	0.05 ± 0.01^a^	0.32 ± 0.00^b^	0.02 ± 0.00^a^	5.06 ± 2.02^b^	12.15 ± 0.23^c^	125.75 ± 3.37^a^
XV	0.89 ± 0.62^ab^	0.63 ± 0.06^a^	0.13 ± 0.03^c^	86.06 ± 21.19^c^	50.60 ± 12.51^ad^	17.09 ± 4.46^b^
*F*-ratio	2.522	1.922	2.09	30.498	13.42	6.966
*P*-value	0.018	0.069	0.049	0.000	0.000	0.000
**October**						
I	0.27 ± 0.17^a^	0.50 ± 0.38^b^	0.04 ± 0.02^a^	10.43 ± 3.61^b^	33.13 ± 17.66^a^	135.32 ± 81.65^a^
II	0.10 ± 0.01^c^	0.86 ± 0.01^a^	0.01 ± 0.00^c^	8.41 ± 0.00^b^	11.45 ± 0.01^ac^	119.11 ± 1.53^b^
III	0.15 ± 0.08^ac^	0.62 ± 0.38^ab^	0.03 ± 0.01^a^	12.18 ± 9.72^b^	20.74 ± 18.80^ac^	47.99 ± 38.44^c^
IV	0.60 ± 56^b^	0.35 ± 0.33^b^	0.07 ± 0.02^ab^	13.04 ± 4.38^b^	48.25 ± 21.05^a^	111.75 ± 20.01^ab^
V	0.69 ± 0.01^b^	0.44 ± 0.01^b^	0.04 ± 0.00^a^	1.59 ± 0.01^c^	40.76 ± 0.01^ab^	60.46 ± 3.46^c^
VI	0.57 ± 0.36^ab^	0.52 ± 0.34^b^	0.04 ± 0.03^ab^	11.49 ± 11.42^b^	58.54 ± 12.09^b^	75.56 ± 32.24^b^
VII	0.25 ± 0.15^a^	0.43 ± 0.32^b^	0.03 ± 0.02^a^	16.29 ± 9.53^b^	27.67 ± 16.55^a^	30.70 ± 26.11^c^
VIII	0.22 ± 0.07^a^	0.51 ± 0.32^b^	0.03 ± 0.01^a^	14.19 ± 10.91^b^	24.99 ± 21.53^a^	56.11 ± 25.16^c^
IX	0.82 ± 0.25^b^	0.59 ± 0.12^b^	0.07 ± 0.01^b^	67.37 ± 5.15^a^	50.94 ± 15.87^a^	6.43 ± 6.77^e^
X	0.25 ± 0.11^a^	0.87 ± 0.19^ab^	0.03 ± 0.01^a^	18.08 ± 9.80^b^	63.32 ± 0.01^b^	23.31 ± 7.99^d^
XI	0.21 ± 0.00^a^	0.10 ± 0.02^c^	0.02 ± 0.00^a^	10.71 ± 0.00^b^	9.85 ± 0.01^ac^	134.55 ± 0.65^a^
*F*-ratio	3.997	0.703	2.059	9.196	2.433	10.374
*P*-Value	0.001	0.716	0.052	0.000	0.021	0.000

*P < 0.05 in one-way ANOVA. Different letters indicate significant differences between types at the 0.05 significance level. TN, total nitrogen content; TP, total phosphorus content; SOM, soil organic matter; Moist, soil moisture content; Alt, altitude; Dis, distance from water.*

**FIGURE 3:**
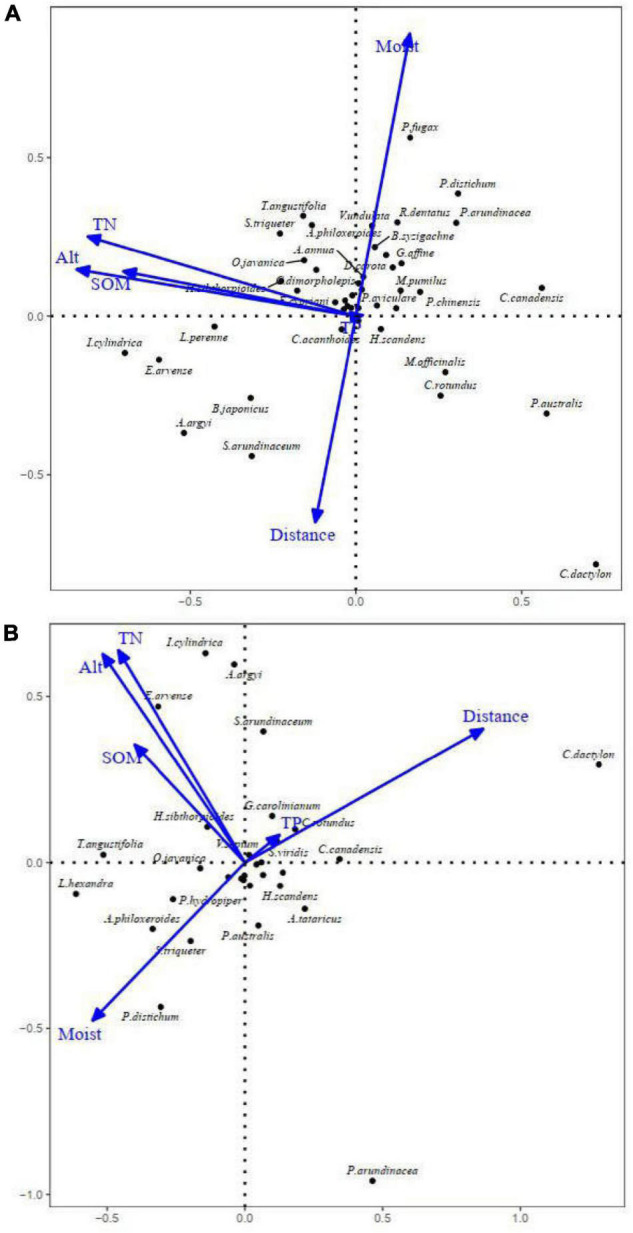
Redundancy analysis (RDA) ordination diagrams of the frequently observed species in quadrats along the middle and lower reaches of the Hanjiang River. **(A)** RDA ordination diagram of 131 quadrats in June 2019; **(B)** RDA ordination diagram of 134 quadrats in October 2019.

The RDA ordination diagrams for the frequently observed species were presented in Figures 3A,B. The position of the species in the ranking map roughly reflected the ecological environment suitable for the species. The distribution of species in June and October was basically the same. *I. cylindrica*, *Equisetum arvense*, and *Lolium perenne* tended to grow in areas with high soil nutrient content, high soil organic matter and high altitudes. *P. fugax* (which just emerged in June), *P. distichum* and *T. angustifolia* were found in areas with moist soil. *C. dactylon*, *S. arundinaceum*, *B. japonicus*, and *Cyperus rotundus* were distributed far from the water and exhibited low nutrient dependence. The distribution of species was consistent with the dominant species community.

**FIGURE 4:**
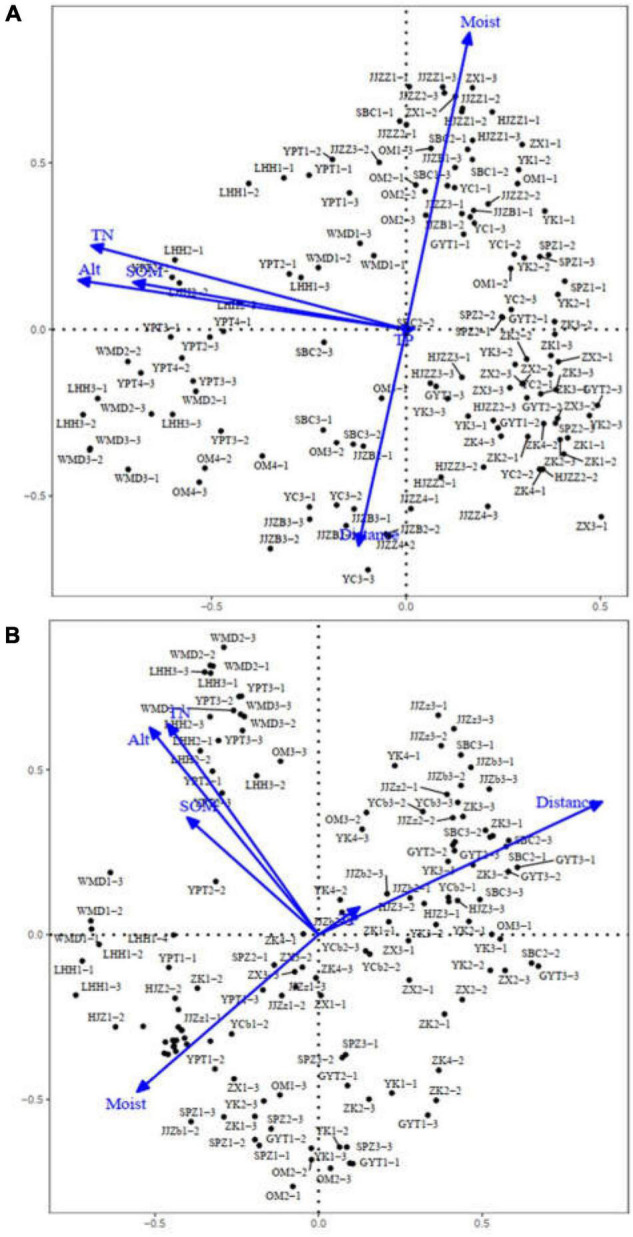
Redundancy analysis (RDA) ordination diagrams of quadrats and environmental factors along the middle and lower reaches of the Hanjiang River. **(A)** RDA ordination diagram of 131 quadrats in June 2019; **(B)** RDA ordination diagram of 134 quadrats in October 2019.

Using RDA, the herbaceous plant community species and environmental factors of 131 and 134 quadrats in June and October, respectively, were analyzed to explore the relationship between different quadrats and environmental factors, as shown in Figures 4A,B. Transects of the sampling sites were consistent with the water gradient and varied from transect 1 to transect 4 along the water gradient and with distance from the river. This result was consistent with TWINSPAN classification, which reflected the environmental gradient of different vegetation types in the riparian zone. Typical samples upstream of the dams, such as WMD and LHH, and other samples downstream of the dams occupied different locations. Compared with Alt and soil nutrients (such as TN and SOM), Moist and Dis were the two most important factors influencing vegetation types.

## Discussion

### Vegetation Distribution and Environmental Factors

This study investigated the middle and lower reaches of the Hanjiang River at a large scale, examining the riparian vegetation distribution pattern across all of the natural ecological environment. The vegetation types were similar to those of the Yangtze River Basin, heavily comprised of species in the families Gramineae and Compositae (Fan et al., 2017). Gramineae contains many common dominant plants in wetland habitats (Xie et al., 2015). Compositae taxa often exhibit rapid evolution and can adapt to riparian habitats by means of reproductive dominance and resistance to stress (Liu et al., 2021; Zheng et al., 2021). The herbaceous plants observed, such as *P. distichum*, *P. arundinacea*, and *T. angustifolia*, were typical hydrophytes of the Yangtze River Basin and highly dependent on water (Sharma et al., 2008; Liao et al., 2010; Li et al., 2015; Guechi and Benabdesselam, 2020; Noyszewski et al., 2021). The communities at higher altitudes and with lower soil water contents were mainly composed of *I. cylindrica*, which was consistent with the Yangtze River Basin (Wang et al., 2009; Su et al., 2013). Apart from the vegetation types found in our investigations, the unique vegetation types of the middle and lower reaches of the Hanjiang River include *T. angustifolia*, *A. argyi*, and *P. australis*, which have characteristics typical of species in the region (Yang F. et al., 2012).

Redundancy analysis results showed that Moist, TN, Alt, SOM, and Dis had significant effects on species and quadrat distributions. As expected, the soil water content was inversely proportional to the Dis, and the shorter the Dis, the higher the soil water content. Moist was one of the most important factors affecting plant colonization of riparian ecosystems (Lou, 2011; Sueltenfuss et al., 2020). It was well known that almost all plants in nature follow a water gradient (Saha et al., 2016). Alt had been shown to be an important factor affecting the distribution of species along north-to-south rivers (Mallik and Richardson, 2009; Li et al., 2012). The change of Alt caused differences in hydrothermal conditions and affected the physical and chemical properties of soil, thus shaping the distribution of species (Chambers et al., 2004). In addition to Moist and Alt, plant community types were also affected by TN and SOM. In a study conducted at Poyang Lake in the same region, TN was found to be one of the most important factors affecting plant community types (Wang et al., 2014). Soil nutrients were dominant factors in the succession process of ecosystems and environmental change (Li et al., 2012). The hydrodynamic and water deposition conditions of rivers affected the physical and chemical composition of soil and determine the morphology, structure and fertility of plants. Thus, the structure, dynamics and distribution of species were closely related to environmental characteristics (Campos and Souza, 2002).

### Effects of Cascade Dams on Vegetation Types

The portion of the middle and lower reaches of the Hanjiang River from Wangfuzhou Dam to Danjiangkou Dam was mainly utilized for power generation and navigation. The construction of the Cuijiaying and Yakou Dams was mainly intended to reduce shipping pressure on the Hanjiang River after the water diversion from the south to the north (Song et al., 2018). A large number of studies have found that the Danjiangkou Dam has greatly changed the hydrology of the middle and lower reaches of the Hanjiang River (Wang et al., 2015; Yu et al., 2020; Zhang et al., 2020; Zhao et al., 2021). The main manifestations of this change included the following three issues. First, the total flow of the river has dropped sharply. For example, compared with before the start of water transfer in 2014, the extreme minimum flow of Huangjiagang hydrological station (near sample point YPT), Huangzhuang hydrological station (near sample point ZX) and Xiantao hydrological station (near sample point GYT) increased by 52, 57, and 63%, respectively, and the average maximum flow decreased by 74, 75, and 67%, respectively (Yu et al., 2020). Second, the dams substantially changed the water level. To ensure the navigation conditions of the middle and lower reaches of the Hanjiang River, the reservoir area upstream of the cascade dams has been maintained at a high water level for a long time, and the water level has small inter- and intra-annual changes. The river below the dams has been in a state of chronic water shortage (Zhang et al., 2020). Third, there have been changes in extreme water conditions. The time of extreme low water levels has changed, while the time of extreme high water levels has decreased (Wang et al., 2015). Changes in the hydrological regime result in increases in water area and the inundation frequency upstream of the dam, while decreasing the fluctuation range in the riparian area (Baena-Escudero et al., 2021). However, the river downstream of a dam becomes a dry riverbed under long periods of water shortage. Thus, the construction of dams changed the environment of the river flowing up and down the dam, the flow mechanism of the river, and the habitat structure of the riparian zone (Merritt and Wohl, 2010).

Some studies have shown that the influence of dams upstream is mainly associated with the inundation caused by the rising water level after dams’ operation, some non-submerged areas being replaced by submerged areas and terrestrial plants being replaced by hygrophytes (Merritt and Wohl, 2010; Li et al., 2012). Hygrophyte and flood-tolerant species that can withstand longer floods thrived in the reservoir area upstream of the dams. *T. angustifolia* was a typical species of communities upstream of the dams in the middle and lower reaches of the Hanjiang River both in June and October (Table 1). This perennial aquatic to semiaquatic herb is more tolerant of deep water than other *Typha* species, with a plant height of 1.5–3 m (Bendix et al., 1994; Brix, 1994; Saha et al., 2016; Pieper et al., 2018; Hong et al., 2020). This species is widely distributed across Yangtze floodplain lakes (Yang et al., 2019). Unlike the habitats of the Three Gorges Dam riparian area in the same basin, which was mainly composed of annual and small hygrophic plants (Yi et al., 2020), the riparian areas upstream of the dams in the middle and lower reaches of the Hanjiang River were flooded year-round and the habitat conditions were more stable. Many wetlands with water levels higher than naturally occurring reference wetlands are invaded and dominated by species of *Typha* (Rigotti et al., 2021). Some studies have found that after dams are built, river ecosystems evolve from riparian to lacustrine owing to the formation of the reservoir areas upstream of dams (Nilsson et al., 2002; Xu and Milliman, 2009; Moura et al., 2011). The construction of dams has made the habitats upstream of dams similar to lake ecosystems, and large hygrophytes, such as *T. angustifolia*, were more likely to develop as dominant species (Merritt et al., 2010; Perbiche-Neves et al., 2011). On the other hand, we found that the vegetation types of the second and third transects of the sample sites were fixed with small seasonal differences and mainly dominated by perennial xerophytes such as *I. cylindrica* and *A. argyi*. The main reason for this pattern may be the water diversion of the Danjiangkou Reservoir, resulting in water shortages in the middle and lower reaches (Yu et al., 2020). In order to maintain normal navigation in the middle and lower reaches, the reservoir area is always maintained at a fixed water level with only small fluctuations in water level. Therefore, the formation and renewal of the upland plant community in the riparian zone by natural disturbances, such as floods, were reduced, and the community type gradually became less diverse and more xeromorphic (Lytle and Poff, 2004).

Downstream of dams, owing to the interception of water by dams, the riparian zone that was previously scoured by short-term seasonal floods experienced a quick withdrawal, thus forming a large tidal flat (Morais et al., 2009; Stratimirovic et al., 2021). This change has provided living spaces for seasonal pioneer species (Oja et al., 2003). For example, the dominant annual herbaceous plants *B. japonicus*, *P. fugax*, and *E. canadensis* were observed in some plots in June. The first transects downstream of the dams near the water in the middle and lower reaches of the Hanjiang River were mainly composed of *P. arundinacea* and *P. distichum*, which are common dominant species in rivers under monsoon climates (Middleton et al., 1991; Valk et al., 1993). Large perennial hygrophytes cannot survive flooding, and smaller hygrophytes thus become the dominant species. At the same time, owing to long-term water shortages, the habitat of upland areas that are less affected by flood gradually changed from hygrophytic to xerophytic, and the highly drought-tolerant *I. cylindrica* can thus easily become the dominant species (Brewer, 2008; Tan et al., 2010). Owing to changes in hydrological conditions, *I. cylindrica* and *P. distichum* often alternate as the dominant species in transition zones from waterfronts to highlands (Liao et al., 2010). At the same time, the original vegetation of the whole basin is lost because of the rapid drop of water level and the long exposure time of tidal flats, consistent with the succession of hygrophytic communities to xerophytic communities.

## Conclusion

Soil moisture, altitude, soil TN and distance from the water were the main environmental factors affecting plants in the riparian zone of the middle and lower reaches of the Hanjiang River. Soil moisture was the most important factor leading to the zonal distribution of communities along the waterside to upland. After the construction and operation of cascade dams in the middle and lower reaches of the Hanjiang River, the continuities of vegetation along the riparian zones were destroyed, which can be divided into two habitats: upstream and downstream of the dam. The riparian zone near the water upstream of the dam was dominated by large aquatic plants, and the upland area was dominated by xerophytes. The community types gradually became less diverse. The riparian zone near water downstream of the dam was dominated by perennial herbs, and the highland area was also dominated by xerophytes. The community type gradually shifted to xerophytic. Therefore, after the operation of cascade dams, measures, such as proper release of water in the reservoir area and enhancement of the positive effects of floods, should be taken to protect and restore fragmented and degraded habitats.

## Data Availability Statement

The raw data supporting the conclusions of this article will be made available by the authors, without undue reservation.

## Author Contributions

JY and E-HL conceived the study, designed the experiments, and supervised the entire study. JY, E-HL, CY, and YX participated in the field survey. JY completed the laboratory experiments and wrote the manuscript. All authors contributed to the article and approved the submitted version.

## Conflict of Interest

The authors declare that the research was conducted in the absence of any commercial or financial relationships that could be construed as a potential conflict of interest.

## Publisher’s Note

All claims expressed in this article are solely those of the authors and do not necessarily represent those of their affiliated organizations, or those of the publisher, the editors and the reviewers. Any product that may be evaluated in this article, or claim that may be made by its manufacturer, is not guaranteed or endorsed by the publisher.

## Footnotes

1
http://www.iplant.cn/foc/

2
http://www.R-project.org
